# Statistical analyses in disease surveillance systems

**DOI:** 10.1186/1753-6561-2-s3-s7

**Published:** 2008-11-14

**Authors:** Andres G Lescano, Ria Purwita Larasati, Endang R Sedyaningsih, Khanthong Bounlu, Roger V Araujo-Castillo, Cesar V Munayco-Escate, Giselle Soto, C Cecilia Mundaca, David L Blazes

**Affiliations:** 1 US Naval Medical Research Center Detachment (NMRCD), Lima, Peru; 2 Universidad Peruana Cayetano Heredia, Lima, Peru; 3 US Naval Medical Research Unit #2 (NAMRU-2), Jakarta, Indonesia; 4 National Institute of Health Research and Development, Jakarta, Indonesia; 5 National Institute of Hygiene and Epidemiology, Ministry of Public Health, Vientiane, Lao PDR; 6 General Directorate of Epidemiology, Ministry of Public Health, Lima, Peru

## Abstract

The performance of disease surveillance systems is evaluated and monitored using a diverse set of statistical analyses throughout each stage of surveillance implementation. An overview of their main elements is presented, with a specific emphasis on syndromic surveillance directed to outbreak detection in resource-limited settings. Statistical analyses are proposed for three implementation stages: planning, early implementation, and consolidation. Data sources and collection procedures are described for each analysis.

During the planning and pilot stages, we propose to estimate the average data collection, data entry and data distribution time. This information can be collected by surveillance systems themselves or through specially designed surveys. During the initial implementation stage, epidemiologists should study the completeness and timeliness of the reporting, and describe thoroughly the population surveyed and the epidemiology of the health events recorded. Additional data collection processes or external data streams are often necessary to assess reporting completeness and other indicators. Once data collection processes are operating in a timely and stable manner, analyses of surveillance data should expand to establish baseline rates and detect aberrations. External investigations can be used to evaluate whether abnormally increased case frequency corresponds to a true outbreak, and thereby establish the sensitivity and specificity of aberration detection algorithms.

Statistical methods for disease surveillance have focused mainly on the performance of outbreak detection algorithms without sufficient attention to the data quality and representativeness, two factors that are especially important in developing countries. It is important to assess data quality at each state of implementation using a diverse mix of data sources and analytical methods. Careful, close monitoring of selected indicators is needed to evaluate whether systems are reaching their proposed goals at each stage.

## Background

Most analyses performed with data from disease surveillance systems focus on establishing baseline disease rates and testing outbreak detection algorithms [1,2]. Another outcome commonly evaluated is the timeliness of data reporting [3]. However, the performance of disease surveillance systems also needs to be evaluated and monitored during other stages of surveillance implementation. For example, outbreak detection algorithms often need to adapt to systematic variations in the frequency of conditions under surveillance [4]. Therefore, it is meaningful to understand and incorporate the seasonal or day-of-week variability in the caseload in order to implement outbreak detection algorithms that can better respond to these variations. Thus, a diverse set of data collection processes and statistical analyses should be implemented and continuously used to monitor and evaluate surveillance systems.

In developing countries, surveillance is often conducted in the context of more limited resources than in developed settings, frequently lacking appropriate computer systems and laboratory diagnostic capabilities, and without sufficient numbers of well-trained physicians [5]. Syndromic surveillance has thus emerged as an alternative to the lack of physicians and laboratory diagnostics, and in some cases is based on monitoring the frequency of patients' signs and symptoms instead of relying on clinical or laboratory-confirmed diagnosis [6]. The introduction of syndromic surveillance in developing countries has also met with increased need for more comprehensive statistical analysis of the information generated, as less is known about the characteristics and behavior of the data streams used in these novel surveillance approaches. We present a brief overview of selected key statistical procedures proposed for different stages of the implementation of epidemiological surveillance systems. Proposed analyses have been classified according to three implementation stages: planning, early implementation and consolidation. Specific emphasis is placed on statistical analyses needed for syndromic surveillance systems implemented in resource-limited settings aiming at early warning and outbreak detection.

The statistical procedures proposed in this paper were developed and applied between 2000 and 2006 during the planning stages and implementation of Alerta [7] and the Early Warning Outbreak Response System (EWORS) [8], two electronic early warning surveillance systems currently in place in resource-limited settings of Asia and the Americas. Alerta monitors clinical diagnosis of mandatory-reporting conditions twice per week within the Peruvian Navy and Army since 2003, and is currently being expanded to other countries in the Americas. Alerta is implemented by the U.S. Naval Medical Research Center Detachment (NMRCD), Peru and uses technology of Voxiva S.R.L. EWORS monitors daily the signs and symptoms of patients with conditions of potential infectious origin in sentinel hospitals, and is implemented in both Southeast Asia and in Peru. EWORS was developed in 1999 by the U.S. Naval Medical Research Unit #2 (NAMRU-2) in Indonesia, and implemented in collaboration with the Ministries of Health in each country. Numeric examples are drawn from the experiences of Alerta in Peru and EWORS in Indonesia, Lao PDR and Peru.

## Proposed statistical analyses by stage

### Planning stage

To demonstrate the feasibility of conducting surveillance, often it is important to prove that surveillance will not place a burden on healthcare personnel, and that timely data can be generated with existing resources. This is particularly the case for syndromic surveillance systems that use data that otherwise are not collected routinely. Pilot tests of the data gathering forms of proposed new surveillance systems should be conducted in advance (with >30 patients, ideally), in order to measure the **mean data collection and entry time, **(i.e., time needed to obtain information from a patient and to input data in a computer). Averages and standard deviations are commonly estimated, and for Alerta and EWORS data collection takes <1 minute per case and does not distract personnel or patients. **Data distribution time**, on the other hand, estimates the time required to send data from the surveillance site to the central hub, usually via the Internet or by phone. It can be estimated beforehand or can be recorded automatically by the system itself, and usually takes <15 m for EWORS (daily) or Alerta (twice per week), showing that little connectivity time is needed, and that dial-up connections are sufficient in most surveillance sites.

### Early implementation stage

**Figure 1:**
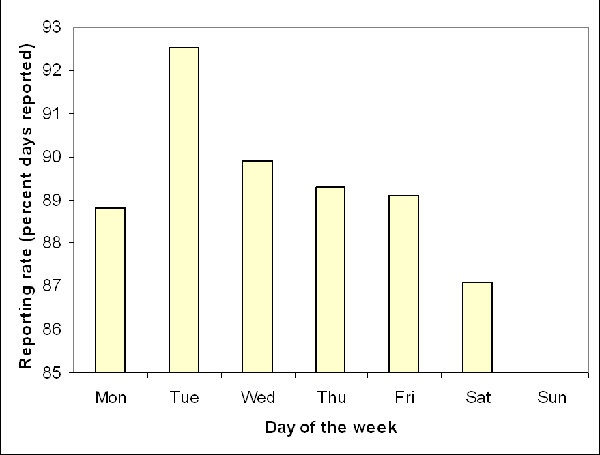
Average daily reporting rates (percent days with data reported) across EWORS sites in Peru, 2005–2006. Surveillance sites do not open on Sundays.

Once surveillance is implemented, **reporting rates estimated as the percentage of days with reported data **(Table 1) should be routinely monitored at least until surveillance sites reach acceptable reporting rates (often >80%). In Peru, reporting rates are monitored weekly for Alerta and EWORS, and these analyses showed that EWORS, a daily-reporting surveillance system, presents substantial day-of-week variations in reporting rates, ranging from an average of 87% on Saturdays to 92% on Tuesdays (Figure 1). We consider that weekly monitoring of reporting rates can provide an appropriate balance between effort and data quality, at least early in the implementation stage of a surveillance system. Sites with low reporting rates (overall or localized in some specific days of the week) usually receive additional oversight and re-training, as chances of outbreak detection can be impaired by poor data quality [9]. Timeliness is also crucial during the early implementation stage, and its relevance has been previously discussed in detail [3].

**Table 1:** Average and range of reporting rate (percent days with data reported) across sites, EWORS 2000 – 2006.

**Country**	**Overall percent reporting rates (range)**
Indonesia	96 (91 – 100)
Lao PDR	93 (88 – 94)
Peru*	89 (69 – 100)

* Surveillance of signs and symptoms, conducted from Monday to Saturday only.

**Completeness of reporting (percent of all eligible subjects whose data was actually recorded in the surveillance system) **assesses the representativeness of the data reported. Estimating this indicator often requires a labor-intensive process involving sporadic site visits to manually evaluate the fraction of patients who visited a surveillance site seeking medical attention and were actually included in the surveillance system. EWORS and Alerta sites evaluated in Peru showed completeness rates ranging from 66% to 90% (Araujo-Castillo R, personal communication 2007).

**Figure 2:**
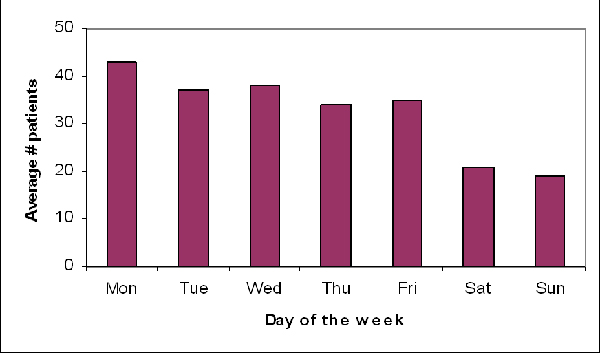
Average number of cases surveyed by day of the week, EWORS Lao PDR 2003–6.

Once a steady, satisfactory level of reporting completeness and timeliness is achieved, **the caseload and patient profile should be described **(Table 2) to better understand the population under surveillance and identify features potentially relevant for outbreak detection. The patient profiles in small district clinics can differ substantially from cases seen at reference hospitals, and important differences can also be observed between surveillance in children versus adult populations. Each of these subpopulations can also help to identify different types of outbreaks. Similarly, analyses of patient profiles could show that cases in certain surveillance sites travel frequently, which could result in higher risk of disease introduction in those sites. This would therefore require more careful, intensified surveillance for potential aberrations. Finally, analysis of patient profiles can also point out sources of systematic variability in the caseload that need to be addressed by outbreak detection algorithms, especially those acting on short periods such as day-of-week effects (Figure 2).

**Table 2:** Main sociodemographic characteristics of cases surveyed, EWORS 2000 – 2006.

**Characteristic**	**Indonesia**	**Lao PDR**	**Peru**
Male (%)	55.6	52.6	52.3
Median age (years)	7	14	3
Traveled recently (%)	0.1	6.1	2.5

**Patients per day**	**27**	**33**	**4**

### Consolidation stage

**Figure 3:**
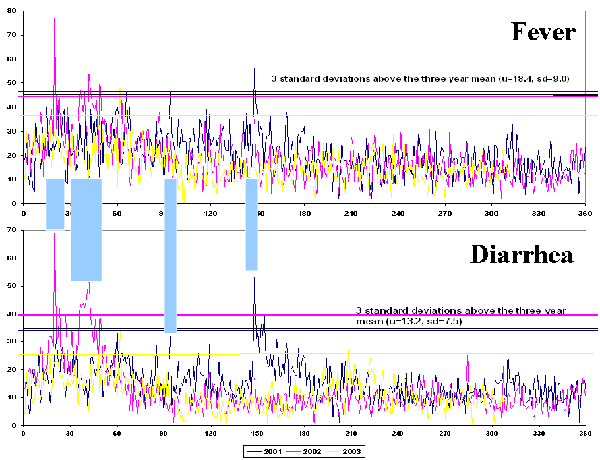
Daily number of cases of fever and diarrhea showing simultaneous increases, EWORS Indonesia, 2001 – 2003.

Syndromic surveillance often allows the evaluation of large numbers of health conditions or combinations of them. For example, EWORS collects 29 signs and symptoms in South East Asia and 33 in Peru, which we have observed can be present in hundreds of combinations. Monitoring that many symptom combinations every week is unmanageable and impractical. In situations like this, where too many symptom combinations or other potential outcomes exist and can be surveyed, **data analyses can target a few carefully selected outcomes to monitor routinely **in order to minimize false positive alerts and avoid an unnecessary burden on public health professionals. For example, during the patient profile analyses conducted with EWORS data, three clearly defined combinations of symptoms (that we will refer to as 'syndromes') consistently accounted for nearly all cases reported in different countries (Table 3). This finding limits the need to monitor the nearly countless numbers of other possible combinations of symptoms. Some combinations of febrile, respiratory and gastroenteric syndromes may be more strongly correlated, clinically relevant and frequent, deserving special attention and probably requiring to be analyzed as a single entity. For example, observations of multiple simultaneous spikes in the frequency of fever and diarrhea in one Indonesian site (Figure 3) suggest the occurrence of outbreaks of infectious diarrhea. A more direct approach to deal with multiple possible symptoms and their combinations is to define and monitor the occurrence of these 'syndromes' as individual entities. Alternatively, a more sophisticated approach could use multivariate methods to analyze each individual symptom (data stream) and adjust for the correlation between symptoms.

**Table 3:** Most frequent symptoms and syndromes and percent patients affected, EWORS, 2000 – 2006.

**Symptom/syndrome**	Indonesia	Lao PDR	Perú
**Most frequent symptoms**			
First	Fever (71)	Fever (75)	Fever (80)
Second	Cough (52)	Cough (44)	Cough (63)
Third	Cold (46)	Headache (37)	Rhinorrhea (36)
Fourth	Diarrhea (17)	Cold (27)	Malaise (31)
Fifth	Vomiting (16)	Malaise (25)	Sore throat (25)
			
**Most frequent syndromes**			
Febrile	71	75	80
Respiratory*	47	51	70
Gastroenteric**	34	25	31
Any of these three	96	89	96

The numbers represent the percent of patients with the symptom or syndrome, respectively

* Cough or sore throat, ** Diarrhea or vomiting.

As this article places specific emphasis on early warning outbreak detection surveillance systems, one of the expected outcomes of such systems will often be the actual detection of an aberration or potential outbreak. **The performance of outbreak detection algorithms is measured by their sensitivity **(percent detected of all outbreaks), **specificity **(often expressed by the average time between warnings) and **detection timeliness **(delay between outbreak onset and detection). Numerous algorithms exist and abundant literature describe their performance [6,10], although with known limitations: 1) performance assessments still rely on simulations instead of 'real' outbreaks, 2) measuring 'true' performance requires outbreak investigations and parallel detection systems as a gold standard, and 3) evidence from developing countries remains remarkably limited.

## Discussion

Researchers conducting statistical analyses applied to disease surveillance systems often place more interest on outbreak detection algorithms [2,4,6], describing in substantially less detail the systems' performance, data quality and the epidemiological profile of the population under surveillance. Surveillance conducted in resource-limited settings, however, often suffers from low reporting coverage and data completeness, which in some cases may be insufficient to support accurate, timely outbreak detection. These operational issues must be addressed before the performance of the system as a whole can be assessed. Although our analyses and evidence are limited to developing country settings, surveillance systems in more developed countries would probably also benefit from increased analysis of data quality and system performance beyond aberration detection.

Outbreak detection algorithms should match the characteristics of the surveillance data available, and only a careful analysis of existing surveillance data may reveal unique features that need to be addressed. A few examples include the presence and magnitude of day-of-week effects, their effect in comparison with seasonal variation, the most frequent disease outcomes among the population, and the socio-demographic units within the population.

Surveillance in the context of developing countries is unique in many aspects. Few surveillance systems currently exist, and entire sub-groups of populations such as the military are often excluded [5]. The implementation of new systems such as Alerta and EWORS provides an opportunity to look critically at the surveillance system evaluation framework and expand the current arsenal of evaluation procedures. As current pandemic threats [11] and international regulations demand more extensive surveillance [12], the evaluation of new systems should begin earlier on their implementation in order to enhance their overall chances of success.

## Conclusion

Statistical methods for disease surveillance have focused mainly on the performance of outbreak detection algorithms and have not paid sufficient attention to the data quality and representativeness, two factors that are especially important in developing countries. Whether the final endpoint of surveillance is outbreak detection, situational awareness, or estimation of trends, these aims cannot be accomplished without adequate intermediate outcomes such as reporting coverage, data quality and completeness. We advocate the use of a more holistic approach to statistical analyses in which indicators relate to the entire surveillance process. Assessment of data quality using a diverse mix of data sources and analytical methods is key during each stage of its implementation. Careful, close monitoring of selected indicators is also crucial to evaluate whether systems are reaching their proposed goals at each stage. A more balanced, diverse analysis of surveillance systems data is essential in the current context, as new surveillance systems are implemented in response to pandemic threats and the recently updated international health regulations.

## Competing interests

The authors declare that they have no competing interests.

## Authors' contributions

AGL led the preparation of the manuscript and its revisions. All authors participated in the implementation of surveillance systems. Specifically, RPL, ERS and KB participated in the implementation of EWORS in Indonesia and Lao PDR, AGL, RA, GS, CCM and DLB participated in the implementation of EWORS and Alerta in Peru; and CVM worked in the implementation of EWORS in Peru. All authors participated in the preparation and review of the manuscript.

## Disclaimers

The views expressed in this article are those of the authors and do not necessarily reflect the official policy or position of the Ministries of Health or Governments of Peru, Indonesia or Lao PDR, the U.S. Department of the Navy, the U.S. Department of Defense, nor the U.S. Government.

Several authors of this manuscript are employees of the U.S. Government. This work was prepared as part of their duties. Title 17 U.S.C. §105 provides that 'Copyright protection under this title is not available for any work of the United States Government'. Title 17 U.S.C. §101 defines a U.S. Government work as a work prepared by military service member or employee of the U.S. Government as part of that person's official duties.
